# Phylogeographic support for horizontal gene transfer involving sympatric bruchid species

**DOI:** 10.1186/1745-6150-1-21

**Published:** 2006-07-27

**Authors:** Nadir Alvarez, Betty Benrey, Martine Hossaert-McKey, Andrea Grill, Doyle McKey, Nicolas Galtier

**Affiliations:** 1 Laboratoire d'Entomologie Evolutive, Université de Neuchâtel, 11 rue Emile-Argand, 2007 Neuchâtel, Switzerland; 2 Centre d'Ecologie Fonctionnelle et Evolutive, CNRS, 1919 route de Mende, 34293 Montpellier Cedex 5, France; 3 Laboratoire Génome Populations Interactions Adaptation, CNRS, Place Eugène Bataillon, 34095 Montpellier Cedex 5, France

## Abstract

**Background:**

We report on the probable horizontal transfer of a mitochondrial gene, *cytb*, between species of Neotropical bruchid beetles, in a zone where these species are sympatric.

The bruchid beetles *Acanthoscelides obtectus*, *A. obvelatus*, *A. argillaceus *and *Zabrotes subfasciatus *develop on various bean species in Mexico. Whereas *A. obtectus *and *A. obvelatus *develop on *Phaseolus vulgaris *in the Mexican Altiplano, *A. argillaceus *feeds on *P. lunatus *in the Pacific coast. The generalist *Z. subfasciatus *feeds on both bean species, and is sympatric with *A. obtectus *and *A. obvelatus *in the Mexican Altiplano, and with *A. argillaceus *in the Pacific coast. In order to assess the phylogenetic position of these four species, we amplified and sequenced one nuclear (*28S rRNA*) and two mitochondrial (*cytb*, *COI*) genes.

**Results:**

Whereas species were well segregated in topologies obtained for *COI *and *28S rRNA*, an unexpected pattern was obtained in the *cytb *phylogenetic tree. In this tree, individuals from *A. obtectus *and *A. obvelatus*, as well as *Z. subfasciatus *individuals from the Mexican Altiplano, clustered together in a unique little variable monophyletic unit. In contrast, *A. argillaceus *and *Z. subfasciatus *individuals from the Pacific coast clustered in two separated clades, identically to the pattern obtained for *COI *and *28S rRNA*. An additional analysis showed that *Z. subfasciatus *individuals from the Mexican Altiplano also possessed the *cytb *gene present in individuals of this species from the Pacific coast. *Zabrotes subfasciatus *individuals from the Mexican Altiplano thus demonstrated two *cytb *genes, an "original" one and an "infectious" one, showing 25% of nucleotide divergence. The "infectious" *cytb *gene seems to be under purifying selection and to be expressed in mitochondria.

**Conclusion:**

The high degree of incongruence of the *cytb *tree with patterns for other genes is discussed in the light of three hypotheses: experimental contamination, hybridization, and pseudogenisation. However, none of these seem able to explain the patterns observed. A fourth hypothesis, involving recent horizontal gene transfer (HGT) between *A. obtectus *and *A. obvelatus*, and from one of these species to *Z. subfasciatus *in the Mexican Altiplano, seems the only plausible explanation. The HGT between our study species seems to have occurred recently, and only in a zone where the three beetles are sympatric and share common host plants. This suggests that transfer could have been effected by some external vector such as a eukaryotic or viral parasite, which might still host the transferred fragment.

**Reviewers:**

This article was reviewed by Eric Bapteste, Adam Eyre-Walker and Alexey Kondrashov.

## Open peer review

Reviewed by Eric Bapteste, Adam Eyre-Walker and Alexey Kondrashov. For the full reviews, please go to the Reviewers' comments section.

## Background

The traditional view of evolution supports that DNA is transferred vertically from parent to offspring. Hybridization and genetic transfer between different species is usually strongly limited. However, exceptions to this rule, i.e. the horizontal transmission of genetic material between distantly related organisms (HGT), are increasingly recognized as an important process of evolution in prokaryotes [1-4]. In eukaryotes, however, reported cases of between-species genetic exchanges not involving hybridization have been essentially limited to noncoding or parasitic transposons or virus sequences. Exceptions to this are a few instances of horizontal transfer of selected genes between prokaryotes and eukaryotes (e. g., [5-7]) and two reports suggesting lateral transfer of mitochondrial genes between genetically distant land plant groups [8,9]. No instance of HGT in animals has been reported up to now.

In a recent paper, Martin [10] discusses the relevance of the HGT hypothesis in eukaryotes, focusing on land plants, and stresses the need for corroborating studies in other eukaryote groups. However, he addresses the necessity of careful interpretation of HGT data, and points to the requirement of first discussing such data in the light of standard theories of vertical inheritance of genes.

**Figure 1:**
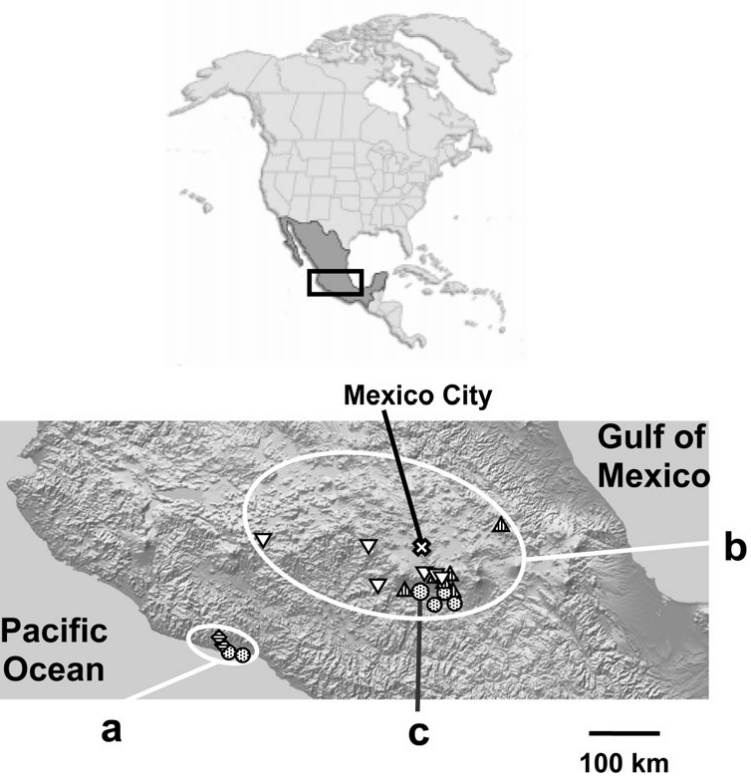
Geographic origin of *Acanthoscelides obtectus *(), *A. obvelatus *(▽), *A. argillaceus *() and *Zabrotes subfasciatus *() individuals sampled. **(a) **Pacific coast populations; only one *cytb *gene is present in *Z. subfasciatus *populations (SJB, ELA). **(b) **Altiplano populations; two *cytb *genes are present in the same individual in some *Z. subfasciatus *(OCM, YAU, TLA); **(c) **population of origin of the *Z. subfasciatus *laboratory colony.

Our study investigates the phylogenetic relationships between four Neotropical bruchid beetles (Coleoptera, Bruchidae), namely *Acanthoscelides obtectus *Say, *A. obvelatus *Bridwell, *A. argillaceus *Sharp, and *Zabrotes subfasciatus *Boheman. These beetles develop on seeds of several bean species (genus *Phaseolus*): *Acanthoscelides obtectus *and *A. obvelatus *feed on the common bean (*P. vulgaris *L. group), *A. argillaceus *on the Lima bean (*P. lunatus *L. group), and *Z. subfasciatus *on both. As *A. obtectus*,*A. obvelatus *and *Z. subfasciatus *can develop on beans of the same species, they are often sympatric in wild or cultivated common beanpopulations. We sampled individuals from these three species in the southern part of the Mexican Altiplano, one such zone of sympatric occurrence of *A. obtectus*,*A. obvelatus *and *Z. subfasciatus *(Figure 1). Although the geographic range of *A. argillaceus *overlaps with those of its two congeners, it is rarely found in the same habitat, because of its distinct host plant. *Acanthoscelides argillaceus *and the generalist *Z. subfasciatus*, however, commonly co-occur in wild Lima bean populations [11]. This is the case in the Pacific coast of Mexico (Figure 1), where individuals from these two species were sampled.

The genera *Acanthoscelides *and *Zabrotes *are morphologically very different [12], and are thought to have diverged during the Palaeocene (65 to 54.8 mya) or the Eocene (54.8 to 33.7 mya) [13], whereas the three *Acanthoscelides *species studied here belong to the same morphologically defined group within the genus and seem to have diverged during the Miocene (23.8 to 5.3 mya) [14,15]. To confirm the phylogenetic position of these four species in the context of host plant adaptation (see [16]), we performed amplification and sequencing of two mitochondrial genes and one nuclear gene.

## Results

**Figure 2:**
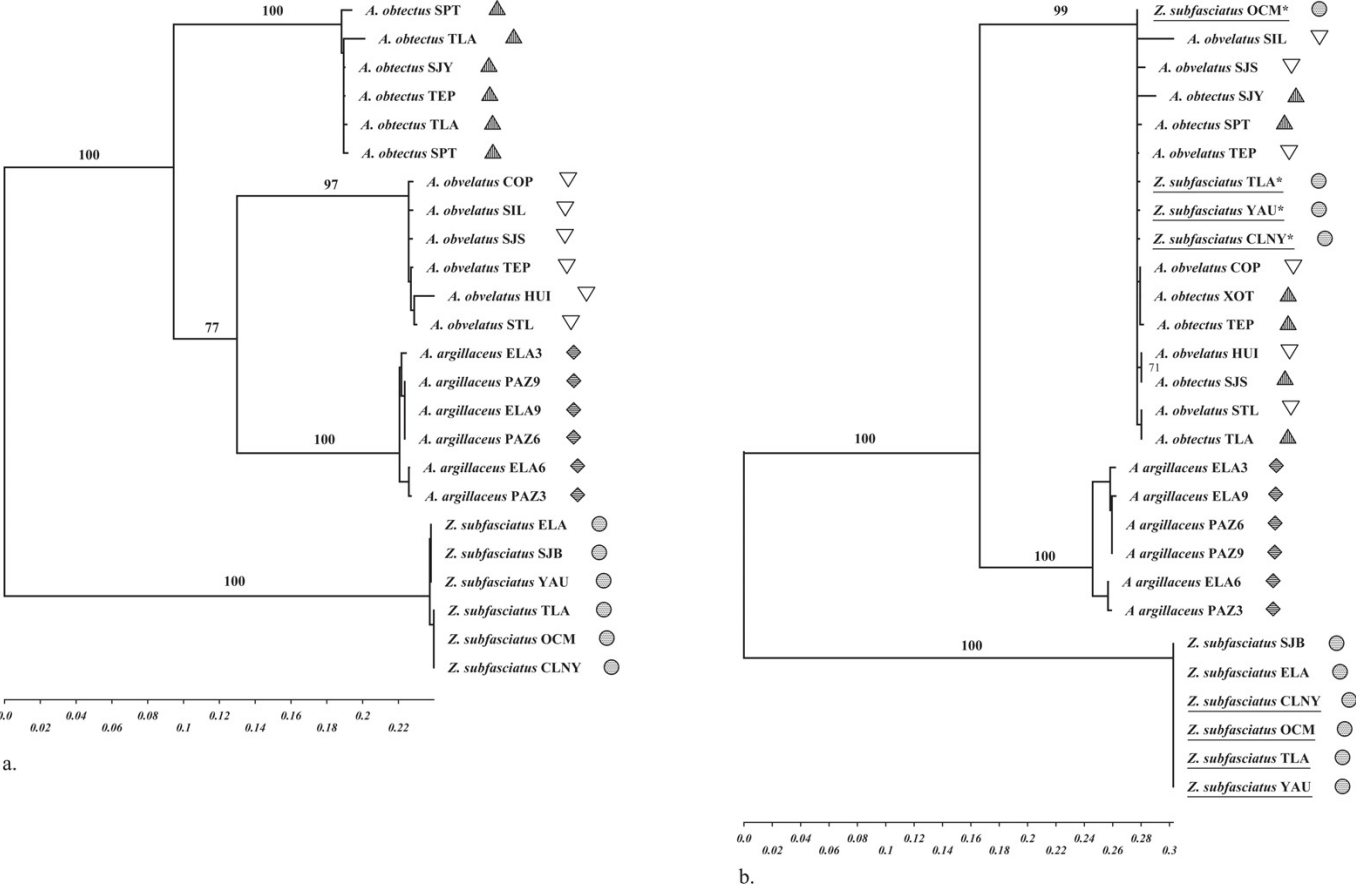
Phylogenetic patterns. **a**. Phylogenetic relationships between different populations of *Acanthoscelides obtectus *(), *A. obvelatus *(▽), *A. argillaceus *() and *Zabrotes subfasciatus *() for the mitochondrial *COI *gene. An identical topology is supported by *28S rRNA *(not shown); **b**. Phylogenetic relationships between different populations of *Acanthoscelides obtectus *(), *A. obvelatus *(▽), *A. argillaceus *() and *Zabrotes subfasciatus *() for the *cytb *gene. Underlined individuals correspond to *Z. subfasciatus *populations from the Mexican Altiplano – some carry two *cytb *haplotypes. Stars (*) indicate *Z. subfasciatus *haplotypes identical to haplotypes from *Acanthoscelides*. "*Z. subfasciatus *CLNY" corresponds to an individual of the laboratory colony affected by HGT.

For each of the three studied genes, different models of evolution were selected by likelihood ratio tests: for *COI*, the Tamura-Nei model with a proportion of invariable sites and a gamma distribution was selected; for *cytb*, the Hasegawa-Kishino-Yano model with a gamma distribution was selected; for *28s rRNA*, the Kimura 2-parameters model was selected. Topologies of phylogenetic trees were congruent for *28S rRNA *and *COI*, and showed each species as a monophyletic group, the three *Acanthoscelides *species forming a distinct clade from *Z. subfasciatus *(see Figure 2a) (*28S rRNA *[accession numbers AY881176–AY881195, DQ152235–DQ152238]; *COI *[accession numbers AY881196–AY881214, AY881232, DQ152239–DQ152242]). *COI *genetic distances ranged from 0.273 to 0.324 substitutions per site between *Acanthoscelides *spp. and *Zabrotes subfasciatus*, and from 0.172 to 0.212 substitutions per site among *Acanthoscelides *species. According to a recent calibration of a beetle mitochondrial molecular clock [17], such genetic distances correspond to divergence times of 35–40 My between *Acanthoscelides *spp. and *Zabrotes subfasciatus*, and 20–25 My among *Acanthoscelides *species. These divergence times were confirmed by estimates obtained from mitochondrial *12S rRNA *[16], and are fairly similar to the fossil record [13].

The analysis of *cytb *sequences, in contrast, revealed an unexpected pattern (see Figure 2b) (accession numbers AY422474–AY422479, AY422485–AY422488, AY881215–AY881232). First, individuals of *A. obtectus *and *A. obvelatus *were not separated in two distinct clades, but grouped into a single, little variable monophyletic unit, as would be expected for individuals belonging to the same species. A core clade containing 46 of the 50 sequenced individuals from the two species included 14 closely related haplotypes (maximal distance: 0.0084), some of them shared by the two species, others private to *A. obtectus *or *A. obvelatus*. In contrast, *A. argillaceus *individuals clustered in a separate clade, whose mean distance to the *A. obtectus *– *A. obvelatus *core clade was 0.143. Secondly, *cytb *data revealed a surprising discrepancy among the individuals of *Z. subfasciatus*. Whereas an expected 0.25 nucleotide divergence from *Acanthoscelides *spp. was found for the *Z. subfasciatus *individuals from the Pacific coast of Mexico, three *Z. subfasciatus *individuals from the Altiplano (coming from three different populations) showed a *cytb *sequence identical to the most common *A. obtectus *– *A. obvelatus *core clade haplotype.

PCR with specific primers and subsequent sequencing revealed that the three Altiplano *Z. subfasciatus *individuals surprisingly carried both *cytb *genes (Figure 2b), their own plus the one demonstrating an *Acanthoscelides *origin. The specific primers were then applied to other *Z. subfasciatus *individuals from the Altiplano and the Pacific coast. In the Altiplano, 20 individuals out of 70 were revealed to carry both *cytb *genes – while the remaining 50 only carried their own specific *cytb *– whereas in the Pacific coast, all the 40 sampled individuals were revealed to carry only their own *cytb *haplotype. The specific primers were then also applied to 25 individuals of a laboratory colony of *Z. subfasciatus *that had been maintained for 10 months in the Laboratory of Evolutionary Entomology at the University of Neuchâtel (Switzerland). The colony was founded using individuals from the Mexican Altiplano, i.e., potentially affected by the HGT. These individuals had not been in contact with any *Acanthoscelides *for about 5–10 generations. Three individuals out of the 25 were revealed to carry both *cytb *genes, while 22 showed only the *Z. subfasciatus *haplotype. The number of individuals sampled in each site and the number of individuals fitting the core clade haplotype are summarized in Table 2.

An analysis of *cytb *sequence variation within the *A. obtectus *– *A. obvelatus *core clade revealed no frameshift, and a low (0.106) ratio of non-synonymous to synonymous changes, significantly lower than 1 (likelihood ratio test: 2(ln [Likelihood(M1)]-ln [Likelihood(M2)]) = 47.5; χ^2 ^[1 df]: P < 10-4), indicating that this gene is under selection. Furthermore, these sequences included no mitochondrial stop codon, but typically five nuclear stop codons, strongly suggesting that this gene is replicated by a mitochondrion, and neither by a nuclear nor a bacterial machinery.

## Discussion

The *cytb *pattern shown by *A. obtectus*, *A. obvelatus *and three Mexican Altiplano *Z. subfasciatus *individuals is highly incongruent with traditional taxonomy, and with the *COI *and *28S rRNA *pattern. This is even more astonishing, considering that *cytb *and *COI *are supposedly genetically linked in the non-recombining mitochondrial genome.

Four hypotheses can be addressed to explain this unexpected result: (i) experimental contamination, (ii) hybridization, (iii) pseudogenisation and (iv) horizontal gene transfer.

### Contamination hypothesis

All experiments were simultaneously conducted on many (>200) bruchid samples, including museum specimens with low amounts of DNA, by the same researcher in the same laboratory. Only the *cytb *PCR for Mexican Altiplano individuals yielded an additional, unexpected band. In *Acanthoscelides*, no individual from the Pacific Coast *A. argillaceus *carried the additional gene, although they were analyzed simultaneously with the *A. obtectus *and *A. obvelatus *samples. A putative laboratory contaminant would not only have affected the samples coming from the Mexican Altiplano but also individuals from other sites and particularly museum specimens, which contain very small amounts of DNA, given the fact that samples were randomized on the 96-well plates, both during DNA extraction and PCR reactions. Another strong argument against the contamination hypothesis is that several individuals of the core clade from both *A. obtectus *and *A. obvelatus *demonstrated private *cytb *haplotypes. These haplotypes cannot be the consequence of a contamination, since they were found only once (we sequenced all the amplified *cytb *fragments). In the case of a contamination, a single haplotype would be expected to occur in a large number of random samples, whereas our data show many related but distinct – and sometimes private – haplotypes found in a non-random subset of the samples. Sampling contamination (e.g., the presence of *Acanthoscelides *tissues in *Z. subfasciatus *samples) can also be excluded, since three *Z. subfasciatus *individuals sampled from the experimental colony showed the additional *cytb *PCR band. The experiments on colony individuals (DNA extraction, PCR) were conducted several weeks after the analysis of the wild samples, making any cross-experiment contamination impossible.

### Hybridization hypothesis

Hybridization between *Acanthoscelides *species and *Zabrotes subfasciatus *is very unlikely. First, the split between the two genera dates back to around 40 mya (molecular clock data) to 60 mya (fossil record) ago. Moreover, they demonstrate a very high level of nucleotide divergence (25%). Usually, the degree of genetic divergence in documented cases of interspecific hybridization in insects never exceeds 5%-10% of nucleotide divergence for protein-encoding mitochondrial genes [18-20], which is much lower than the 25% divergence found between *Acanthoscelides *and *Zabrotes*. Secondly, hybridization appears functionally impossible, given the highly differentiated morphology, especially in the male genitalia. The lateral lobes of the male genitalia – which play the decisive role of pushing aside parts of the female genitalia to allow penetration by the median lobe, and which are known to be part of a specific lock/key system – are spectacularly different between the two genera. Borowiec [12] points out that the structure of the aedeagus is very different in *Zabrotes *and *Acanthoscelides *and highly specialized in both. The lateral lobes are particularly distinct, short and almost entirely fused in *Zabrotes*, but elongate and divided by a deep cleft almost to the base in *Acanthoscelides*. In the bruchid family, in which many cryptic species remain to be described, male genitalia are often the only character to distinguish between otherwise morphologically identical (but never hybridizing) sibling species. This clearly explains why any attempt to produce hybrid offspring between *Acanthoscelides *and *Zabrotes *failed (N. Alvarez, unpublished observations). Moreover, even in the two morphologically similar *Acanthoscelides obtectus *and *A. obvelatus*, no evidence of interspecific hybridization has been detected *in natura*, despite the genetic analysis (by means of diagnostic microsatellite loci) of hundreds of individuals from the two species, among which dozens originated from sympatric populations [15].

### Pseudogene hypothesis

An additional surprise in our data is that the putatively transferred fragment appears to evolve under purifying selection. The rate of non-synonymous substitutions is significantly lower than expected under a model involving a non-selected gene, and there is no insertion or deletion in any *cytb *sequenced fragment. Moreover, the fragment seems to be expressed in a mitochondrion. Given that the gene is under purifying selection, it could hardly have been translated using the nuclear genetic code, since in that case, five codons would have been translated in stop-codons. These properties argue for an active gene, rather than a pseudogene. A pseudogene – which by definition can hardly ever be under purifying selection – would not show a bias to synonymous over non-synonymous mutations, and its sequence would most likely show nucleotide insertion or deletion. Furthermore, in the exceptional context of a selected pseudogene [21], the gene must not contain any stop codon when translated with the nuclear genetic code. The combined evidence suggests that genes from the core clade are (i) under purifying selection and are therefore not pseudogenes, and (ii) are translated in a mitochondrion.

### Horizontal gene transfer hypothesis

Our data clearly argue against any standard explanation for the genetic pattern we observed. A horizontal transfer of the *cytb *gene between *A. obtectus *and *A. obvelatus*, and from one of these species to *Z. subfasciatus*, seems the most plausible hypothesis, even though the probability of such a mitochondrial genetic exchange between distinct eukaryote species is very low (but is invoked in a recent study in plants [8,9]). We assume that species polyphyly in the *cytb *core clade results from a relatively ancient HGT process having started at the time of the core clade ancestor or earlier. Alternatively, one may interpret this clade as resulting from a series of numerous, very recent HGT events. Under the latter hypothesis, the functional constraints we outlined in the last paragraph would have pre-dated HGT's, and would not guarantee that the transferred gene is active and expressed in a mitochondrion.

The data available do not allow formal rejection of this latter hypothesis, under which the transferred pieces of DNA could even be nuclear pseudogenes. This hypothesis requires, however, a sudden, very recent increase of the HGT rate from zero to a very high value. This could be conceivable in the context of a rapid, infectious process (if, for instance, one specific strain of a parasite acquires the ability to switch species and invade the new niche), but in this case one would expect to see a single transferred haplotype (or a small number of them), whereas the intricate phylogeny of the core clade implies several events of HGT involving several distinct haplotypes.

Under either of these two hypotheses (one ancient HGT or several recent ones), the transfer from one species to another could possibly have been carried out by an external vector. Candidate vectors include viruses, prokaryotes such as *Wolbachia *or other endosymbiotic bacteria (a case of horizontal transfer of genes from *Wolbachia *to the nuclear genome of its host was recently reported in bruchids [22]) or even eukaryotes (e.g., intra- or extra-cellular parasites, endoparasitoïds). The location of the transferred *cytb *in the recipient species remains to be investigated. A parsimonious hypothesis is that the transferred *cytb *is still hosted by its vector. A eukaryotic parasite might have "domesticated" a bruchid mitochondrion (or at least some bruchid mitochondrial gene), carrying it on when switching to a new species. Such an hypothesis could explain the presence of two *cytb *genes in *Z. subfasciatus *individuals from the Altiplano, suggesting that one gene could be carried by a parasite, and the other could belong to the mitochondria of *Z. subfasciatus *itself. This could also be the case in *A. obtectus *or *A. obvelatus*, even though we could not yet demonstrate the existence of a second *cytb *gene in individuals belonging to the core clade.

If we consider that the vector may belong to a eukaryotic group (i) capable of mitochondrial recombination and (ii) showing a genetic code that uses the same mitochondrial stop-codons as those used by the mitochondria of invertebrates – these two conditions are for example filled by most unicellular eukaryotes – then the mitochondria carried by the parasite may have "included" the "bruchid"-*cytb *in its genome after recombination. Another interesting point is that the transferred gene replicates in *Z. subfasciatus*, since it survived ~5–10 generations in the laboratory.

### Ecological context of HGT

As this HGT event has been detected only between individuals from the Altiplano where the three species co-occur and share the same host plant, it gives clues to the ecological context which may favor such a gene exchange. Indeed, the ecologically distinct *A. argillaceus *does not demonstrate a HGT pattern for *cytb*, despite its phylogenetic proximity to *A. obtectus *and *A. obvelatus*. This suggests that the probability of the occurrence of lateral gene transfer is partly controlled by the environment. The occurrence of HGT between bruchid species (i) presenting a high degree of phylogenetic divergence but (ii) sharing some dimensions of their ecological niche (e.g., their host plant) could indicate that ecology, as well as phylogeny, might play a role in the distribution of genetic variation across taxa.

## Conclusion

Recent horizontal gene transfer (HGT) between *A. obtectus *and *A. obvelatus*, and from one of these species to *Z. subfasciatus *in the Mexican Altiplano, seems the most plausible hypothesis to explain the pattern we observed in our data for *cytb*. The transfer could have been effected by some external vector such as a eukaryotic parasite, which might still host the transferred fragment.

Obviously, additional molecular, cellular and tissular characterization would be of great relevance to the understanding of this intriguing system, of the exact length and structure of the apparently transferred fragment, and the nature of the genome hosting it. The contemporaneous nature of this putative HGT – it is still polymorphic in *Z. subfasciatus *at least – makes it a promising model for further investigation of the mechanisms underlying genetic exchanges between species

## Methods

Individuals of *A. obtectus*, *A. obvelatus*, *A. argillaceus*, and *Z. subfasciatus *were sampled in the southern Mexican Altiplano. Coordinates of sampled sites are given in Table 1. We amplified and sequenced bi-directionally two mitochondrial genes – cytochrome oxidase I (*COI*) and cytochrome b (*cytb*) – and one nuclear gene – 28S ribosomal rRNA (*28S rRNA*) – from 6 to 25 individuals in each species, using universal primers (*28S rRNA*: 28ee and 28 mm; *COI*: C1-J-2183 and TL2-N-3014; *cytb*: CB-J-10933 and CB-N-11367 [23]). Total genomic DNA was extracted using DNeasy™ 96-well kit (QIAGEN). PCR amplifications were performed in a final volume of 10 μL, which contained 1 μL of extracted DNA, 1 μL of 25 mM MgCl2, 0.1 μL of 10 mM dNTPs, 1 μL of PCR buffer (Eurogentec), 1 unit of Taq DNA polymerase (Eurogentec Red Goldstar™), 0.5 μL of forward primer, and 0.5 μL of reverse primer. PCRs were performed separately for each primer pair on a PTC-200™ thermocycler using the following cycling conditions: initial denaturation at 92°C (1 min 30 s); 30 cycles of 92°C (30 s), annealing at 55°C (45 s), 72°C (1 min 30 s); final elongation at 72°C (10 min). Sequencing reaction was carried out using Applied Biosystems BygDye™ protocol. Products of the sequencing reactions were then analyzed on an ABI Prism 310 sequencer. Chromatograms were manually corrected using Chromas 2.23 (Technelysium Pty. Ltd., Helensvale, Australia) and further aligned using ClustalW 1.83 [24]. The best-fit substitution model was determined using Modeltest 3.06 [25] through hierarchical likelihood ratio tests. Phylogenetic trees were reconstructed by maximum likelihood using Treefinder [26]. Distances between groups were determined by distance methods using Mega 2.1 [27] with standard parameters (Kimura 2-parameters; gamma shape parameter = 0.5). Primers specific for *cytb *consensus haplotypes of *A. obtectus *and *A. obvelatus*, and for *cytb *haplotypes of Pacific coast *Z. subfasciatus*, respectively, were designed. Sequences of specific primers to the most common haplotype shared by *A. obtectus *and *A. obvelatus *are: TTGATAACGCAACCTTAACC (forward primer) and GATTAGCAGGAATGAAGTTG (reverse primer). Sequences of specific primers to Pacific-coast *Z. subfasciatus cytb *are: GAGATAATGCAACATTAACA (forward primer) and GGTTTGCGGGCGTAAAATTA (reverse primer). To test whether the sequenced *cytb *genes were under purifying selection (i.e., they were not the result of a nuclear mitochondrial pseudogene), we compared rates of synonymous and non-synonymous substitutions. Two models of codon evolution [28] were tested using PAML 3.14b [29]. In model M1, the ratio of non-synonymous to synonymous evolutionary rate (ω) was set to 1, as expected for a pseudogene. In model M2, the ratio was free to vary. A likelihood-ratio test was performed to compare the two models.

## Reviewers' comments

### Reviewer's report 1

#### Eric Bapteste, Dalhousie University, Halifax, Canada

This paper presents potentially interesting data and a possibly compelling result – lateral gene transfers of genes between animal species-, that if confirmed, would certainly deserve to be reported to a broad audience of readers. Yet, a great deal of caution is required before accepting the conclusion proposed here. In my view the present study is still too weak to support the conclusion.

Certainly, the authors reject several alternative hypotheses to explain the bizarre distribution of *cytb *genes in bruchids. For instance, the presence of two divergent copies of the *cytb *gene in *Z. subfasciatus*, one of them being very conserved in sequence and shared by a set of traditionally distantly related taxa, deserves some explanation. In that respect, the authors are right to attract our attention of this result. They exclude the possibility of contamination, hybridization events and the possibility that one of the copies is in fact a pseudogene. Importantly enough though, I feel that more work is needed to test their final proposition and to conclude in favour of the lateral transfer of the gene between animals, based on the sole evidence presented here. Some more in depth analyses should be performed in order to test their claim and to propose a more precise interpretation of the data. Typically, questions about the mechanism of acquisition of this second copy of *cytb *should be at the heart of such future studies. Nevertheless, some quick adjustments could be incorporated into a moderately revised version of this manuscript.

It might be of some help in deciding if it is premature or legitimate to quote this study as a case of lateral gene transfer in animals if the authors could answer some of the questions below.

1. Is it really impossible that an endoparasite is a source of a contamination, that the relatively conserved *cytb *gene shared by the three species is not of bruchid origin and that the observations may be partly artefactual?

If these species hosted a population of closely related endoparasite, could not two *cytb *sequences be amplified from these organisms? In this case, we would get the sequences of the population of endoparasites (presenting thus some distinct but related haplotypes) and the one for the bruchid? What does a blast of this additional copy teach us: who are the closest completely sequenced relatives having homologues to this additional sequence? Including these data in a broader phylogenetic tree via a broader *cytb *alignment would certainly be a very important additional result to present to the readers. Are these bruchid sequences still monophyletic when a larger taxonomic sample is investigated and, if not, where do these gene copies branch in the tree? How much evidence support that these two sequences are carried in the genome of the bruchid (i.e. in the mitochondrial DNA of the species as the result of an actual transfer)?

##### Response from authors

*The hypothesis of an endoparasite carrying the unusual *cytb *sequences deserves some attention. These sequences, however, are undoubtedly of bruchid origin, since a blast search of Genbank using the additional copy shows that the closest sequences outside *Acanthoscelides *or *Zabrotes *are carried by four bruchids, two *Bruchus *(*B. signaticornis *[AY390728.1]*, B. tristiculus *[AY390729.1]) and two *Bruchidius *(*B. saudicus *[AY625444.1]*, B. rubicundus *[AY625443.1]), all of them found exclusively in the Old World, and therefore unikely to have been in contact with *Acanthoscelides *and/or *Zabrotes *since 90 Mya. Moreover, the first 50 blast hits are all distributed amongst the bruchid family, both in New World genera (i.e*. Acanthoscelides and Zabrotes) *and in Old World genera (i.e*. Bruchus, Bruchidius, Tuberculobruchus, Conicobruchus, Palaeoacanthoscelides and Callosobruchus). *More distant sequences are found in species within the superfamily Chrysomeloidea, which comprises bruchids (e.g*. Anophophora), *or within more distantly related beetles (e.g*. Blackburnia) *or crickets (e.g*. Gryllus). *We therefore believe that presenting a broader phylogenetic tree including sequences from these more distantly-related species would not add useful information, since the additional cytb copy is typical of bruchids, and since the root of the tree is obviously in the *Zabrotes *lineage. Nevertheless, we certainly admit that we cannot be sure by which genome the additional gene is carried; It might be hosted by a eukaryote endoparasite, or by a prokaryotic or viral genome, although we present some evidence for mitochondrial translation. We hope to be able to address this question in a middle-term perspective*.

2. Could insect species of the outgroup carry as well more than one *cytb *copy?

If we admit that the bruchid carries two copies and that this is not contamination, and if it carries them within its mitochondria, the scenario of a transfer is appealing, except if the presence of two copies is a "normal" condition for those insects. Without the study of the genetic composition of the outgroup, it is not clear to me if the apparently original situation of the *Z. subfasciatus *individuals (with two copies of *cytb *present in the Altiplano) is ancestral (vertically inherited) or derived (laterally acquired).

Could it be the case that the outgroup of these species actually already contained two copies of this gene? Are there precedents of related organisms with two *cytb *genes? Could it be tested by studying the composition of some completely sequenced mitochondrial genomes? Could then the present result be explained either (i) by a much more ancient lateral gene transfer (somewhere at the base of the bruchids, in which case the scenarios should try to explain this ancient phenomenon) or if not, eventually (ii) by some complex scenarios of independent genes losses to explain the patchy distribution of *cytb *in current bruchid populations?

##### Response from authors

*In our opinion, the polymorphic nature of this character (i.e. both the ecological/phylogeographic pattern of the distribution of the additional copy and the rare status of *Zabrotes *individuals carrying the two copies within sites) argues for a relatively recent event (i.e., more recent than the basal divergence of all the beetles studied here, as suggested by the reviewer). Even admitting that the outgroup carried two independent copies – that would, for instance, have two distinct functions and be indispensable to these bruchids – a vertical scenario could not have led to such a low (0–1%) divergence between the original *Acanthoscelides *copy and one of those carried by some *Zabrotes subfasciatus *indivudals, knowing that the two species have diverged during more than 50 Mya *[13,30]. *Alternatively, one would have to invoke a very bizarre pattern of gene conversion. We believe that sequence identity in this relatively rapidly-evolving mtDNA gene tends to favour the hypothesis of a relatively recent horizontal transfer over the hypothesis of an ancient divergence of the two copies, followed by vertical double inheritance of both copies*.

3. Can one deduce anything about the mechanisms of recombination that inserted the new copy, if they are all within the mitochondrial bruchid genome?

If there is only one type of mitochondria in the bruchids, and if this mitochondria carries the two gene copies, it seems at first sight that the presence of two *cytb *copies in *Z. subfasciatus *rules out the hypothesis of a legitimate recombination to transfer this gene. Such a mechanism would likely have led to the replacement of one former *cytb *copy by another one, resulting in one copy per species. Could the opposite observation suggest then that the additional *cytb *was transferred in kind of a hitch-hiking process, due to the insertion of one (or more) other markers in the mitochondrial DNA, which could have themselves happened by homologous recombination? In this case the HGT event would be even larger, and would concern several markers. By contrast, for the species presenting only one copy of *cytb*, could we study the location of this single copy with more precision? A (partial) study of the genetic composition of the mitchondrial genome of these bruchids, typically of the genes bordering the *cytb *gene, could be very informative here. They may or may not have retained evidence of recombination or synteny. Would it be possible to characterise the genetic context of the *cytb *genes and test their conservation in later studies to decide if there was one or several instance of LGT and what was their mechanism? Alternatively, could the *cytb *copy be jumping using transposases? Do we have any evidence for/against the implication of transposons in this story?

##### Response from authors

*We agree that sequencing the full mitochondrial genome(s) of bruchid individuals carrying two cytochrome b copies would help understanding further what happened and how. Unfortunately these data are lacking, and are not so easy to collect, given the relatively high level of sequence similarity between the two copies, the lack of knowledge of the genomic structure, and the scarcity of living individuals with two copies – a new round of field sampling is probably required. Sequencing the full mitochondrial genome(s) would also help us in understanding why a "double-pattern" is lacking for other *cytb *-linked genes such as *COI, * which seem to be consistent with the scenario supported by nuclear genes and classical taxonomy*.

4. About the nature of the most likely vector: virus, endoparasite or... even an host reservoir?

I was interested by the different hypotheses presented in this paper regarding the vector of the possible lateral transfer. Viruses and endoparasites might be good candidates indeed, although I was wondering if a third possibility, inspired by the "you are what you eat theory" (see. W.F. Doolittle, 1998), could not be raised as well. Since these bruchid species share a host, could not their host have provided them the additional *cytb *copy?

##### Response from authors

*See our response to question 1*.

5. Ecological scenarios and the interest of harbouring two *cytb *copies to survive in the Altiplano.

I may have misunderstood, but I believe that the authors suggest that the persistence of two copies (or at least the swapping of *cytb *genes) could serve the purpose of an endoparasite. My naïve question is: could not it serve the purpose of the bruchids themselves? Could not they benefit in the Altiplano from such a genomic make up? Do we have any way to investigate the biochemistry/activity of these two divergent copies in more depth that would suggest to us that even if they are related their function is only partially overlapping?

##### Response from authors

*This is an interesting hypothesis. Developing the capacity for research on functional mito-genomics in these non-model animals can only be a very long-term perspective, however*.

### Reviewer's report 2

#### Adam Eyre-Walker, University of Sussex, Centre for the Study of Evolution & School of Biological Sciences, Brighton, United Kingdom

This is an interesting paper which describes what I believe is one of those observations which almost defies rational explanation, although the observation does appear to be real.

##### Response from authors

*We fully agree with this statement*.

In this paper the authors describe a phylogenetic analysis of bruchid beetles from Mexico. They show that for one nuclear and one mitochondrial gene, the species are moderately divergent and phylogenetically well resolved – i.e. each species is monophyletic with high bootstrap support. However, for another mitochondrial gene the pattern is very different; two of the species have an almost identical *cytb *gene which is also shared by some members of the most divergent of the beetle species. All individuals which share this *cytb *sequence come from the same geographical area and live on the same host plant. Intriguingly, those individuals of the species *Z. subfasciatus*, which have the shared *cytb *gene, also have a *cytb *gene which is similar to *Z. subfasciatus *individuals from elsewhere in Mexico; this other *cytb *sequence is as divergent as one might expect given how distantly related this species is to the others.

So how do we explain this bizarre pattern? The authors consider a number of alternatives including contamination, hybridisation and pseudogenisation, none of which seems likely. This leaves horizontal gene transfer as possibly the only other explanation. They suggest that maybe some eukaryotic vector has transferred the *cytb *gene between different species. However, two questions remain. Why has only a part of the mitochondrial DNA been transferred? Individuals from different species share similar *cytb *sequences but have very different mitochondrial *COI *sequences.

##### Response from authors

*See our response to Reviewer 1 (question 3)*.

And why don't all species which have this particular *cytb *sequence also have their own original *cytb *sequence, as individuals of *Z. subfasciatus *appear to have. One can explain these patterns but the explanations are not simple.

##### Response from authors

*This question highlights one limitation of the PCR-only experimental strategy we had in this work. PCR can miss existing gene copies, so that we cannot make sure whether *Acanthoscelides *individuals from the "core clade" carrying the apparently mobile cytb sequence do or do not carry an additional, "private" copy (especially knowing that the mobile copy is probably of *Acanthoscelides *origin). Given the phylogenetic proximity between these two species, we did not succeed in designing specific primers. Again, full-genome data would clarify these questions but are lacking*.

### Reviewer's report 3

#### Alexey Kondrashov, National Institutes of Health, Bethesda, Maryland, United States

The authors claim that they discovered a case of rather recent lateral gene transfer between mitochondrial genomes of moderately related beetles. While the claim is striking, I see no obvious holes in the data and reasoning and, thus, tend to believe it.

A very interesting observation is that *Zabrotes subfasciatus *is polymorphic by presence/absence of laterally acquired *cytb *gene. When this gene is present, it does not replace the original one and, instead, the two coexist within the same mitochondrial genome. This system opens a rare opportunity to study microevolutionary aspects of lateral gene transfer. It would be worthwhile to sequence complete mitochondrial genomes possessing two *cytb *genes (native and foreign).

##### Response from authors

*See our response to Reviewer 1 (question 3)*.

Also, standard selective sweep analysis may tell us whether positive selection favors genomes with the extra, foreign *cytb*. A great system for further analysis of eukaryotic LGT.

##### Response from authors

*This is an interesting suggestion. We analysed separately the sample of "core clade" haplotypes and found significantly (*p <0.05) negative Tajima's D and Fu & Li's F*, which is indicative of a star-like genealogy compatible with the selective sweep (or founder event) hypothesis. Single-locus arguments for selective sweeps are generally weak, however, given the large number of factors potentially influencing the shape of genealogies.

## Authors' contributions

NA carried out most of the molecular genetic studies and drafted the manuscript. BB and AG participated in the writing of the manuscript and revised the final version. MHM and DM contributed in the interpretation of the data and in the writing of the manuscript. Finally, NG supervised data analysis and participated substantially in interpretation of the data and writing of the manuscript. All authors read and approved the final manuscript.

**Table 1:** List of sampled sites.

Code	Site name	Geographic position	Sampled species	Latitude (°North)	Longitude (°West)	Altitude (m)
CLNY	(proceeding from Malinalco)	Altiplano	*Z. subfasciatus*	18°57'13.2"	99°30'08.9"	1935
COP	Copandaro	Altiplano	*A. obvelatus*	19°26'24.6"	101°45'35.5"	2087
ELA	Elabillal	Pacific coast	*A. argillaceus Z. subfasciatus*	18°00'27.0"	102°21'44.8"	28
HUI	Huitzilac	Altiplano	*A. obvelatus*	19°01'24.4"	99°16'23.3"	2544
OCM	Tilapa	Altiplano	*Z. subfasciatus*	19°11'24.5"	99°25'12.2"	1300
PAZ	Playa Azul	Pacific coast	*A. argillaceus*	17°59'20.8"	102°21'14.4"	21
SIL	San Ildefonso	Altiplano	*A. obvelatus*	19°22'19.8"	100°08'56.9"	2400
SJB	San Juan Bosco	Pacific coast	*Z. subfasciatus*	18°07'12.4"	102°08'24.9"	150
SJS	San Jose de los Laureles arriba	Altiplano	*A. obtectus A. obvelatus*	18°58'49.7"	99°00'05.0"	1855
SJC	San Jose de los Laureles abajo	Altiplano	*A. obtectus*	18°58'40.3"	98°58'20.0"	1730
SPT	San Pablo de Tejalpa	Altiplano	*A. obtectus*	18°52'59.8"	99°36'00.3"	1750
STL	Santa Lucia	Altiplano	*A. obvelatus*	18°52'12.5"	100°00'03.7"	1790
TEP	Tepoztlan	Altiplano	*A. obtectus A. obvelatus*	18°59'36.3"	99°07'15.7"	1931
TLA	Tlayecapan	Altiplano	*A. obtectus Z. subfasciatus*	18°57'20.0"	99°03'24.4"	1750
XOT	Xochitlan	Altiplano	*A. obtectus*	19°57'59.9"	97°39'02.0"	1450
YAU	Yautepec	Altiplano	*Z. subfasciatus*	18°45'31.9"	99°01'24.0"	1700

**Table 2:** Number of individuals collected in each site for each species and number of individuals fitting the "core clade" haplotype in each population. In *Zabrotes subfasciatus *populations, all individuals also fitted their own original *cytb *haplotype.

Species	Site	Geographic position	Nb of sampled individuals	Nb of individuals fitting the "core clade" haplotype
*A. argillaceus*	ELA	Pacific coast	6	0
	PAZ	Pacific coast	6	0

*A. obtectus*	SJC	Altiplano	10	10
	SJS	Altiplano	10	10
	SPT	Altiplano	10	10
	TEP	Altiplano	10	10
	TLA	Altiplano	10	10
	XOT	Altiplano	10	10

*A. obvelatus*	COP	Altiplano	10	10
	HUI	Altiplano	10	10
	SIL	Altiplano	10	10
	SJS	Altiplano	10	10
	STL	Altiplano	10	10
	TEP	Altiplano	10	10

*Z. subfasciatus*	CLNY	Altiplano	25	3
	OCM	Altiplano	8	4
	TLA	Altiplano	30	9
	YAU	Altiplano	7	4
	ELA	Pacific coast	20	0
	SJB	Pacific coast	20	0
